# Development and evaluation of an immunochromatographic strip test based on the recombinant UL51 protein for detecting antibody against duck enteritis virus

**DOI:** 10.1186/1743-422X-7-268

**Published:** 2010-10-14

**Authors:** Chanjuan Shen, Anchun Cheng, Mingshu Wang, Kunfeng Sun, Renyong Jia, Tao Sun, Na Zhang, Dekang Zhu, Qihui Luo, Yi Zhou, Xiaoyue Chen

**Affiliations:** 1 Avian Diseases Research Center, College of Veterinary Medicine of Sichuan Agricultural University, Ya'an, Sichuan, 625014, China; 2 Key Laboratory of Animal Diseases and Human Health of Sichuan Province, Ya'an, Sichuan, 625014, China; 3 Epizootic Diseases Institute of Sichuan Agricultural University, Ya'an, Sichuan, 625014, China

## Abstract

**Background:**

Duck enteritis virus (DEV) infection causes substantial economic losses to the worldwide duck-producing areas. The monitoring of DEV-specific antibodies is a key to evaluate the effect of DEV vaccine and develop rational immunization programs. Thus, in this study, an immunochromatographic strip (ICS) test was developed for detecting DEV serum antibodies.

**Results:**

The ICS test is based on membrane chromatography, and uses both the purified recombinant UL51 protein conjugated with colloidal gold and goat anti-rabbit IgG conjugated with colloidal gold as tracers, the purified recombinant UL51 protein as the capture reagent at the test line, and rabbit IgG as the capture reagent at the control line. The specificity of the ICS was evaluated by sera against DEV, Duck hepatitis virus (DHV), Riemerella anatipestifer (RA), Duck E. coli, Muscovy duck parvovirus (MPV), or Duck Influenza viruses (DIV). Only sera against DEV showed the strong positive results. In order to determine the sensitivity of the ICS, anti-DEV serum diluted serially was tested, and the minimum detection limit of 1:128 was obtained. The ICS components, which are provided in a sealed package, require no refrigeration and are stable for 12 months. To evaluate the effect of the ICS, 110 duck serum samples collected from several non-immune duck flocks were simultaneously tested by the ICS test, enzyme-linked immunosorbent assay (ELISA) and neutralization test (NT). The results showed that the sensitivity of the ICS test was almost consistent with ELISA and much higher than NT, has low cost, and is rapid (15 min) and easy to perform with no requirement of specialized equipment, reagent or technicians.

**Conclusions:**

In this work, we successfully developed a simple and rapid ICS test for detecting DEV serum antibodies for the first time. The ICS test was high specific and sensitive for the rapid detection of anti-DEV antibodies, and has great potential to be used for the serological surveillance of DEV infection in the field.

## Background

Duck viral enteritis (DVE) is an acute contagious disease of various types of waterfowl (ducks, geese, and swans) caused by duck enteritis virus (DEV), which is a member of the subfamily Alpha-herpesviridae[1]. The disease affects waterfowl of all ages. Cases of the disease were recorded in domestic ducks in Holland as early as 1923 [2]. In China, the first outbreak of DVE was in 1957 [3]. To date, only a serotype of DEV has been characterized. In duck-producing areas of the world where the disease has been reported, DVE has resulted in significant economic losses in domestic and wild waterfowls due to high mortality, condemnations and decreased egg production [1]. Several studies have indicated that DVE is difficult to monitor and control, because DEV establishes an asymptomatic carrier state in both farmed and wild waterfowl and it is only detectable during the intermittent shedding period of the virus [1,4].

Vaccination has been used as a preventive measure and also for controlling DVE disease outbreaks. Clinical and laboratory tests have confirmed that the attenuated DEV vaccine is an effective biological agents for the prevention and control of DVE, and the monitoring of DEV-specific antibodies is a key to evaluate the effect of the attenuated DEV vaccine and develop the rational immunization programs [5,6]. Rapid and simple test is needed for routine field practice to monitor whether the vaccines have induced antibody to DEV. Generally, the detection of anti-DEV antibodies in the serum samples of ducks usually relies on conventional techniques, such as the neutralization test (NT) [7,8], enzyme-linked immunosorbent assay (ELISA) [9-11], agar gel diffusion test, Dot-ELISA assay, and passive hemagglutination assay [12]. However, the time consuming process, requiring special instrumentations and professional skills would inevitably inhibit these immunoassay techniques from benefiting the poultry farms in field applications. In contrast with these immunoassay methods, immunochromatographic strip (ICS) tests combine chromatography technology with conventional immunoassay to offer an economic, simple and rapid approach for protein analysis and clinical diagnosis, which is especially suitable for a wide variety of field applications even without the use of instruments [13,14]. It has been widely used as an in-field diagnosis tool to detect antibodies [15,16] or antigens [17,18].

The DEV UL51 protein, a conserved tegument protein, is one of 78 putative proteins encoded by the genome of DEV[19-21], and may be involved in virion maturation, similar to other alpha-herpesviruses UL51 proteins described previously [22-24]. Thus, in the present study, based on a recombinant DEV UL51 protein [19], we developed an ICS test for the field detection of DEV serum antibody, and compared the new assay with standard diagnostic tests, ELISA and NT.

## Results

### Preparation and purification of the recombinant UL51 protein

**Figure 1:**
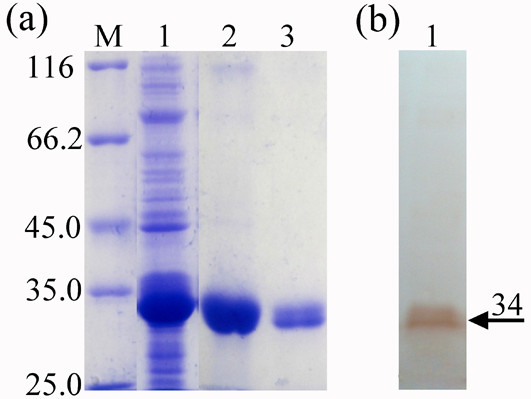
**SDS-PAGE and western blotting analysis of recombinant UL51 protein**. (a) SDS-PAGE analysis of recombinant UL51 protein. Lane M, molecular mass markers (in kDa); lane 1, extract from I mL fermentation cultures of E. coli BL21 (DE3) containing pET28a-UL51 recombinant plasmid; lane 2, the purified recombinant UL51 protein by washing inclusion bodies thrice; lane 3, refolding of inclusion bodies of the purified recombinant UL51 protein by dialyzing. (b) Western blotting analysis of recombinant UL51 protein. Lane 1, western blotting of the purified recombinant UL51 protein, with rabbit anti-DEV antibody and horseradish peroxidase (HRP)-labeled sheep anti-rabbit IgG as the first and second antibody, respectively. The arrowhead indicates the position of recombinant UL51 protein (about 34 kDa).

By the fermenter cultivation, a large number of bacterial cells containing the recombinant UL51 protein were harvest. The recombinant protein obtained was analyzed by SDS-PAGE and western blotting. Coomassie blue staining showed that the UL51 fusion protein was expressed with a molecular mass of approximately 34 kDa (Figure 1a). Western blotting using positive rabbit anti-DEV antiserum as the first antibody demonstrated that the recombinant UL51 protein reacted strongly and specifically with the antiserum raised against DEV (Figure 1b), suggesting that the purified recombinant UL51 protein was suitable as the capture reagent of the ICS.

### Specificity, sensitivity and stability of the ICS test

**Figure 2:**
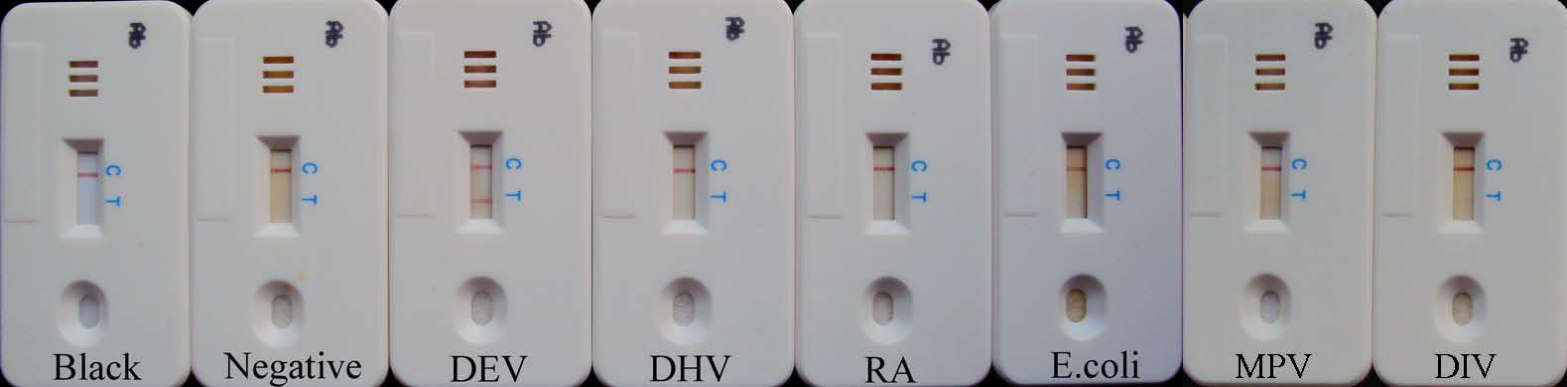
**Specificity of the immunochromatographic strip (ICS) test**. The positive sera against Duck enteritis virus (DEV), Duck hepatitis virus (DHV), Riemerella anatipestifer (RA), Duck E. coli, Muscovy duck parvovirus (MPV), or Duck Influenza viruses (DIV), and sera from healthy ducks were simultaneously tested by the ICS. Similar result patterns were reproduced in repeat experiments (data not shown).

All of the 5 healthy ducks serum samples and 25 standard serum samples positive for other non-DEV pathogens were found negative for anti-DEV antibodies with the ICS test (Figure 2). The results were similar with the blank control which had only one red band at control line (Figure 2). Two bands are seen when 5 standard serum samples positive for DEV was tested (Figure 2). Similar result patterns were reproduced in repeat experiments (data not shown).

**Figure 3:**
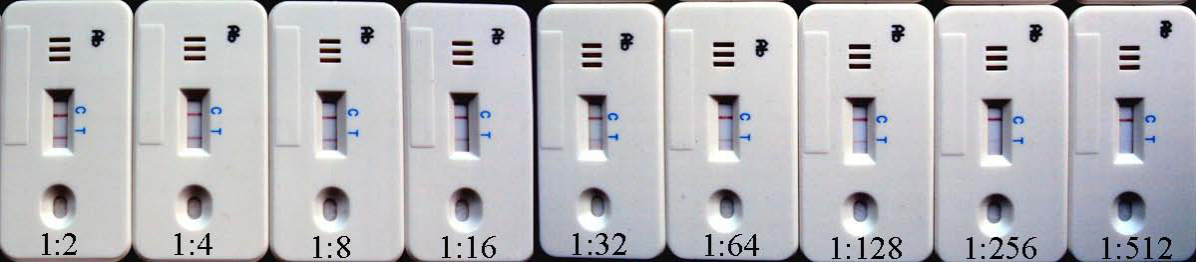
**Sensitivity of the immunochromatographic strip (ICS) test**. Reference positive sera against DEV at different dilutions (from 1:2 to 1:512) were used to analyze DEV-specific antibodies by the ICS test. Data are presented as the mean dilution of each serum at a single assay.

The sensitivity of the ICS was tested with anti-DEV serum diluted serially. Two red bands developed at the test line and control line with a highest dilution of 1:128 (Figure 3). The same results were repeated for 3 times with different personnel. This indicates that the ICS test has a high sensitivity for detecting small amount of anti-DEV antibodies.

With strips being respectively stored for 3, 6, 9, and 12 months at room temperature (about 25°C), all test results were the same from 3 to 12 months, with all known DEV-positive sera being positive and all known DEV-negative sera being negative. False positives were not detected (data not shown).

### Comparison with ELISA and NT

The high sensitivity of the ICS test was also evidenced from the analysis of 110 field serum samples (Table 1). Among the 110 serum samples, 41 samples (37.27%) were positively determined by ICS tests; the percentage of positive sera was comparable to the rate of 42.73% analyzed by the highly sensitive ELISA (P ≥ 0.05), and was notably higher than the 22.73% characterized by NT (P ≤ 0.05). Further analyses revealed that 35 of 41 positive sera samples determined by ICS tests were also positively analyzed by ELISA, while 57 of 69 negative sera were negatively confirmed by ELISA. The ratio of positive and negative consistency for the two methods was 85.37 and 82.61%, respectively (Table 2), with no significant difference in terms of sensitivity between the methods. Compared with NT, 8 of 41 positive sera determined by ICS tests were positively characterized by NT and 52 of 69 negative sera analyzed by ICS tests failed to show positive in NT assays (Table 2). Notably, while very few NT-positive sera were overlooked by ICS tests, many ICS-positive sera, which were confirmed by ELISA, were missed by NT. This suggests that the sensitivity of the ICS test was almost consistent with ELISA and much higher than NT. Importantly, the detection of anti-DEV IgG using the ICS test only took about 15 min; the same serum required a couple of hours with the ELISA assay and more than 3 days with NT.

**Table 1:** Comparison of the percentages of anti-DEV positive sera among ICS, ELISA and NT^a^

	Method		
	
	ICS	ELISA	NT
Positive serum	41	47	25
Negative serum	69	63	85
Ratio of positive^b^	37.27%	42.73%	22.73%

^a ^The total of 110 duck sera samples were simultaneously analyzed by the ICS, ELISA and NT assays.

^b ^The percentiles of anti-DEV positive sera were analyzed by the Chi-square test.

**Table 2:** Comparison of consistency ratios among ICS, ELISA ^a ^and NT^b^

	ELISA		NT	
	Positive	Negative	Positive	Negative
ICS				
Positive	35	6	8	33
Negative	12	57	17	52

^a ^The consistency ratio of the positive number of ICS to the positive number of ELISA is 85.37% and that of the negative number of ICS to the negative number of ELISA is 82.61%.

^b ^The consistency ratio of the positive number of ICS to the positive number of NT is 19.51% and that of the negative number of ICS to the negative number of NT is 75.36%.

## Discussion

As far as we know, the antigen and a specific antibody to it are the two most important components of any serologically diagnostic assay. Generally, because the complex construction of the purified virus may incorporate various host cell proteins, antibodies against expressed protein produced during an immune reaction are more specific than those against purified virus [25]. Moreover, our studies showed that large quantities of recombinant DEV UL51 protein can be produced by large-scale fermentation and purified quickly, but the whole DEV virus cannot be easily produced and purified [26]. Furthermore, in the recent years, ICS test based on a certain recombinant protein [13,15,27], has been widely used for detecting the corresponding anti-virus antibodies. So, the recombinant DEV UL51 protein described in this study, which may be substituted for the whole DEV virus, will no doubt be suitable for ongoing use in the ICS as described above, and will have widespread application in both diagnostic and research work.

In the past few decades, various classical serological methods have been used for detecting antibodies against DEV. The ELISA, which is considered currently the commercial standard for detecting antibody to DEV, uses the purified DEV virions as coating antigen, and is sensitive and specific to antibody against DEV, according to the described previously [10,9]. It can detect large quantities of serum samples with a high sensitivity; however, the ELISA using the whole virus as coating antigen to detect antibodies usually leads to false positives, owing to the complex components of the purified virus, which may incorporate various host cell proteins. Furthermore, ELISA usually requires laboratory operation, skilled technicians, a special instrument, and takes about 3.5 h to complete the measurement, making it difficult for use in the rapid and on-site detection of anti-DEV antibodies. The NT using duck embryo fibroblasts [7], one of the gold standard tests, usually detects antibodies against DEV. This test is very specific, but it has lower sensitivity and commonly takes about 3-5 days to obtain results, and is not suitable for testing large quantities of serum samples. Other methods for detecting anti-DEV antibodies, such as agar gel precipitin test, Dot-ELISA assay, and passive hemagglutination assay [12], are either less sensitive and time-consuming assay, or require special equipments and complex procedures. Therefore, the development of this new, simple and powerful ICS test for the rapid and on-site detection of DEV-specific antibodies is significant.

In this paper, a simple and rapid ICS test based on recombinant UL51 protein has been successfully developed, which could rapidly detect duck IgG antibodies against the UL51 of DEV, both qualitatively and quantitatively, if using serially diluted duck serum, without cross-reaction with antibodies against other tested viruses. In comparison with the commercial standard assay, ELISA, the sensitivity of the ICS test was comparable to the highly sensitive ELISA. Simultaneously, compared with the gold standard assay, NT, the sensitivity of the ICS test was significantly higher than the NT. Unlike these commonly used assays, the ICS test for the detection of DEV-specific antibodies does not require any equipment or skilled technicians and can be conveniently performed on the duck farm by a duck farmer. Importantly, the detection of DEV-specific antibodies by the ICS test only takes about 15 min, which is much faster than the time required for the ELISA and NT assays, and the results can be read directly by the naked eye. Therefore, the ICS test is a high specific and sensitive assay for the rapid and reproducible detection of DEV specific antibodies, which is easy to operate and low in cost. It could be adapted for on-site surveillance in duck flocks.

Outbreaks of DEV throughout the world have resulted in significant economic losses in the duck breeding industry. Effective vaccination to induce immune responses to DEV is expected to control the spread of DVE. Therefore, the epidemiological surveillance of DVE and vaccine-induced immune responses require a sensitive and specific assay that can be conveniently operated to rapidly detect antibodies against DEV. The ICS test has been shown to rapidly detect antibodies to DEV. Its application may economically benefit duck farmer by monitoring the antibody levels of vaccinated duck flocks, and investigating the epidemiology of DEV in unvaccinated duck flocks.

## Conclusions

In summary, we successfully developed a simple and rapid ICS test for detecting DEV serum antibodies for the first time. Compared with the ELISA and NT, the ICS test was able to detect anti-DEV antibodies in naturally infected duck sera with high sensitivity and specificity. The ICS components, which are provided in a sealed package, require no refrigeration and are stable for 12 months. This ICS test is convenient, rapid and easy to perform, with no requirement of specialized equipment, reagent or technicians. Thus, it has great potential to be used for the serological surveillance of DEV infection in the field.

## Methods

### Large-scale preparation and purification of the recombinant UL51 protein

Strain and expression vector: A recombinant expression plasmid pET28a-UL51 was successfully constructed as described previously [19]. Then, the pET28a-UL51 plasmid was transformed into *E. coli *strain BL21 (DE3) (obtained from the Key Laboratory of Animal Disease and Human Health of Sichuan Province). The bacterial cells transformed with the pET28a-UL51 plasmid were grown in Luria-Bertaini (LB) agar medium containing 50 μg/mL kanamycin, and were incubated overnight at 37°C. 200 mL LB medium containing 50 μg/mL kanamycin was inoculated with a freshly grown colony containing the pET28a-UL51 plasmid, and was incubated for 16 h at 37°C as the seed culture.

Fermentation: A twenty liter fermenter (B.Braun, BIOSTATRB, Germany) containing 10 L of LB medium containing 50 μg/mL kanamycin and 1 mL antifoam was inoculated with 2% v/v seed culture (200 mL). 10 L fermentation culture was grown at 640 rpm, 37°C, pH 7.0, and 50% dissolved oxygen (DO) for 2-3 h, until bacterial cells reached the mid-log phase of growth (A_550 nm _= 0.5-1.0). Then the recombinant UL51 protein expression was induced by the addition of 0.4 mmol/L isopropyl-1-thio-β-D-galactoside (IPTG) for 3 h at the same conditions. 1 mL bacterial cultures was taken at 3 h after induction, and the induced bacterial cells were pelleted by centrifugation at 8,000 rpm for 5 min, resuspended in 50 μL of 1 × SDS loading buffer, boiled for 5 min, and analyzed by SDS-PAGE. Then large quantities of bacterial cells were harvested by centrifuging at 8,000 rpm for 10 min and stored at -20°C.

Purification and solution of inclusion bodies: The harvested bacterial cell paste (50.6 g) was resuspended thoroughly in 240 mL of TE buffer (20 mmol/L Tris-HCl, 5 mmol/L EDTA, pH 8.0). The suspension was sonicated for 30-spulses, at least ten times, at 1 min intervals, using a microtip (Branson Ultrasonic Corporation). The pellets of the inclusion bodies were collected by centrifugation at 10,000 rpm for 10 min at 4°C, were resuspended in 120 mL washing buffer (10 mmol/L PBS, 2 mol/L urea, 1% TritonX-100 (v/v), pH 7.4) under constant stirring for 10 min, then followed by centrifugation at 10,000 rpm for 10 min at 4°C, and the above steps repeated twice to release the trapped protein. Finally, the purified inclusion bodies were dissolved in denaturing buffer (10 mmol/L PBS and 8 mol/L urea, pH 7.4) for 1 h at 4°C, and were analyzed by SDS-PAGE.

Renaturation of inclusion bodies: The inclusion bodies were dialyzed in different concentrations of urea buffer solution (6 mol/L, 4 mol/L, 3 mol/L, 2 mol/L, 1 mol/L and 0 mol/L urea in 10 mmol/L PBS, pH 7.4) to refold before determination of the protein content by the Bradford protein assay [28]. The fusion protein solution was adjusted to the concentration of 2 mg/mL, divided into small aliquots, and was analyzed by SDS-PAGE. Rabbit anti-DEV antiserum (obtained from our laboratory) and horseradish peroxidase (HRP)-labeled sheep anti-rabbit IgG were used as the first and second antibody, respectively, for western blotting. The remaining protein solution was stored at -20°C for later use.

### Preparation and assembly of ICS

**Figure 4:**
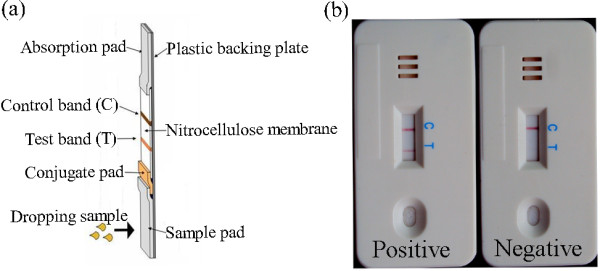
**Schematic diagram of the immunochromatographic strip (ICS) and interpretation of the results detected by the ICS**. (a) Schematic diagram of the ICS. The sample pad, conjugate pad, immobilized nitrocellulose membrane, and absorption pad were glued together on a plastic backing plate. At the test band (T) and control band (C), the purified recombinant UL51 protein of DEV and the rabbit IgG were immobilized, respectively. (b) Interpretation of the results detected by the ICS. A negative result and a positive result were showed in the picture, respectively.

An ICS test for detecting DEV-specific antibodies was developed. A sandwich immunoreaction was performed on the ICS [16,29,30]. Briefly, the ICS assembly consists of a sample pad, a conjugate pad, a nitrocellulose membrane, and an absorption pad. Both the recombinant UL51 protein conjugated with colloidal gold and the goat anti-rabbit IgG conjugated with colloidal gold (provided by Shanghai Goldbio Tech Co., Ltd) were sprayed onto a glass fiber pad. The pad was then dried at 37°C overnight. Purified recombinant UL51 protein, whose optimal concentration was determined as 2 mg/mL, was micro-sprayed onto a nitrocellulose membrane at 1 μL/cm at a position that would become the capture test band (T) of the completed strip. The purified rabbit IgG, whose optimal concentration was determined as 1 mg/mL, was micro-sprayed onto the same nitrocellulose membrane at 1 μL/cm at a position that would become the control band (C); the membrane was dried at 37°C overnight. The conjugate pad was cut into strips 5 mm long and 5 mm wide. The nitrocellulose membrane was sliced into strips 25 mm long and 5 mm wide. One end of the conjugate pad was attached to the sample pad and the other end overlapped the membrane. An absorption pad (cellulose membrane) was attached to the end of the membrane to remove excess reaction mixture. The sample pad, conjugate pad, immobilized nitrocellulose membrane, and absorption pad were glued together on a plastic backing plate (60 mm × 5 mm), as shown in Figure 4a. Each strip was housed in a plastic case that was then stored in a desiccated plastic bag (Shanghai Goldbio Tech Co., Ltd).

### Principle of ICS test

The principle of the ICS test is based on the following theory. If the tested duck serum contains the antibody against DEV, the antibody will be absorbed from the sample pad, which will interact with the recombinant UL51 protein on the conjugate pad to form an antigen-antibody complex. The complex will migrate into the nitrocellulose membrane by capillary action and, subsequently, react with the immobilized recombinant UL51 protein on the testing line (T), generating a red band, the density of which will be in proportion to the concentration of antibody against DEV. Nonreactive goat anti-rabbit IgG on the conjugate pad will run over the test line, and then reacts with the rabbit IgG at the control line (C) of the strip to form the second visible red band. Thus, after approximately 100 μL of the duck serum specimen was added to the sample chamber and let stand for 15 min, the results were considered positive (if the red band was present at both the test line and the control line) (Figure 4b), negative (if the red band appeared only at the control line) (Figure 4b), or invalid (if no red band developed at either lines or only one band appeared at the test line). Evaluation of the test-strip results can be performed with the naked eye and total assay time is less than 15 min.

### Specificity, sensitivity and stability of the ICS test

The specificity of the ICS test was evaluated with standard negative serum samples from 5 healthy ducks, 25 standard serum samples positive for non-DEV pathogens (the pathogens for Duck hepatitis virus (DHV), Riemerella anatipestifer (RA), Duck E. coli, Muscovy duck parvovirus (MPV), or Duck Influenza viruses (DIV)), 5 standard serum samples positive for DEV. All the standard serum samples were supplied by our laboratory.

The sensitivity of the ICS was tested with serially diluted anti-DEV serum. The standard serum sample was diluted 8 times with 10 mmol/L PBS from 1:2 to 1:512. The diluted sera were tested with this ICS. The same procedure was repeated three times with different operators.

The stability of the ICS was determined with the standard positive serum and the standard negative serum. At each sample time, 8 strips that had been respectively stored for 3, 6, 9, and 12 months at room temperature (about 25°C), were tested.

### ELISA

An ELISA for the detection IgG antibody against DEV in serum was performed as previously described [10,9]. In brief, the DEV CHv strain (obtained from our laboratory) virions abundantly propagated in duck embryo fibroblasts (DEF) was purified by differential velocity centrifugation and sucrose density gradient centrifugation. Round-bottomed 96 well polystyrene plates (Nunc MaxiSorp) were coated overnight with the prepared highly purified DEV virions (100 μL/well) at 4°C in a humidity chamber. The plates were washed three times with PBS-T buffer (10 mmol/L PBS containing 0.05% Tween-20), non-specific protein binding sites were blocked with blocking buffer (10 mmol/L PBS containing 1% fetal calf serum) for 60 min at 37°C, and the plates were then washed three further times with PBS-T buffer. A 10-fold dilution series of serum, diluted with PBS, were added and the plates incubated for 60 min at 25°C following by washing, 50 μL of HRP-labeled goat anti-duck IgG (KPL) (1:4000 dilution with PBS containing 1% bovine serum albumin) was added. Following incubation for 60 min at 25°C, the plates were washed and 100 μL 3,3^'^,5,5'-etramethylbenzidine (TMB) substrate solution (KPL) was added along with 0.01% of H_2_O_2 _in 0.05 mol/L citric acid buffer (pH5.0). After 15 min, the reaction was terminated by adding 50 μL of 0.5 mol/L sulfuric acid solution. The absorbance was read at 450 nm on a 96-well plate reader (Model 460, Bio-Rad). The results were expressed as serum antibody titer defined as the log10 of the dilution that generated an optical density (OD) equal to two standard deviations (SD) above the mean background OD of negative control duck sera at 450 nm.

### NT

The NT was performed as previously described [7,8]. Briefly, the serum was heated at 56°C for 30 min to inactivate complement and diluted by means of serial two-fold dilutions in MEM. Then, the diluted sera were equally mixed with a 200 TCID_50 _dose of DEV CHv strain at 37°C for 1 h. The mixtures were inoculated into the DEF cultured in 24-well plates (Corning Incorporated). The cytopathic effect (CPE) was observed, and the neutralizing antibody titer of the serum was calculated using the Reed-Muench formula.

### The analysis of 110 field serum samples

Using the ICS, 110 sera that had been collected from several non-immune duck flocks in Sichuan province, were tested. They were also tested for antibody against DEV using the ELISA and NT following the above instructions.

### Statistical analysis

The percentiles of anti-DEV positive sera were statistically analyzed by Chi-square test and a P value of ≤ 0.05 was considered significantly.

## Competing interests

The authors declare that they have no competing interests.

## Authors' contributions

CJS carried out most of the experiments and drafted the manuscript. ACC, MSW, KFS, RYJ, TS, NZ, DKZ, QHL, YZ, and XYC helped in experiments and drafted the manuscript. All authors read and approved the final manuscript.
